# 3,3′,5-Triiodothyroacetic acid (TRIAC) induces embryonic ζ-globin expression via thyroid hormone receptor α

**DOI:** 10.1186/s13045-021-01108-z

**Published:** 2021-06-26

**Authors:** Huiqiao Chen, Zixuan Wang, Shanhe Yu, Xiao Han, Yun Deng, Fuhui Wang, Yi Chen, Xiaohui Liu, Jun Zhou, Jun Zhu, Hao Yuan

**Affiliations:** 1 Shanghai Institute of Hematology, State Key Laboratory of Medical Genomics, National Research Center for Translational Medicine at Shanghai, Ruijin Hospital Affiliated to Shanghai Jiao Tong University School of Medicine, Shanghai, China; 2 CNRS-LIA Hematology and Cancer, Sino-French Research Center for Life Sciences and Genomics, Ruijin Hospital Affiliated to Shanghai Jiao Tong University School of Medicine, Shanghai, China; 3 Department of Hematology, Sir Run Run Shaw Hospital, Zhejiang University School of Medicine, Hangzhou, China; 4 Université de Paris 7/INSERM/CNRS UMR 944/7212, Equipe Labellisée Ligue Nationale Contre le Cancer, Hôpital St. Louis, Paris, France

**Keywords:** ζ-Globin, Thyroid hormone, Thalassemia, Sickle-cell disease

## Abstract

The human ζ-globin gene (*HBZ*) is transcribed in primitive erythroid cells only during the embryonic stages of development. Reactivation of this embryonic globin synthesis would likely alleviate symptoms both in α-thalassemia and sickle-cell disease. However, the molecular mechanisms controlling ζ-globin expression have remained largely undefined. Moreover, the pharmacologic agent capable of inducing ζ-globin production is currently unavailable. Here, we show that TRIAC, a bioactive thyroid hormone metabolite, significantly induced ζ-globin gene expression during zebrafish embryogenesis. The induction of ζ-globin expression by TRIAC was also observed in human K562 erythroleukemia cell line and primary erythroid cells. Thyroid hormone receptor α (THRA) deficiency abolished the ζ-globin-inducing effect of TRIAC. Furthermore, THRA could directly bind to the distal enhancer regulatory element to regulate ζ-globin expression. Our study provides the first evidence that TRIAC acts as a potent inducer of ζ-globin expression, which might serve as a new potential therapeutic option for patients with severe α-thalassemia or sickle-cell disease.

**Supplementary Information:**

The online version contains supplementary material available at 10.1186/s13045-021-01108-z.

To the editor,

The inherited hemoglobin disorders, including thalassemia and sickle-cell disease, are an emerging global health burden. It is estimated that in excess of 330,000 affected infants are born annually [1]. There is an urgent need to identify new types of agents for these hemoglobinopathies.

Embryonic ζ-globin gene expression is normally limited to the early stages of primitive erythropoiesis and transcriptionally silenced at 6–7 weeks of gestation [2]. Relatively little attention has been paid to understanding the processes that control ζ-globin expression in the past few decades. Intriguingly, recent reports have shown that continued expression of human ζ-globin is not only able to rescue a lethal α-thalassemia mouse model [3], but also efficiently inhibits sickle hemoglobin polymerization in a transgenic mouse model of sickle-cell disease [4], suggesting induction of this embryonic globin may act as a novel therapeutic for both α-thalassemia and sickle-cell disease. However, pharmacologic compounds capable of activating ζ-globin gene expression have not yet been available so far.

3,3′,5-triiodothyroacetic acid (TRIAC, also known as Tiratricol) is a naturally occurring thyroid hormone metabolite, with high affinity for thyroid hormone receptors. It has been used on an empirical basis to treat patients with thyroid hormone resistance [5]. More recently, TRIAC has also displayed great therapeutic potential for the treatment of Allan–Herndon–Dudley syndrome [6]. Although the relevance and use of TRIAC have been extensively explored over the last decades, its role in the regulation of globin gene expression has not previously been elucidated.

As a first step in seeking whether TRIAC affects globin gene expression, we used zebrafish as a model organism, which is an ideal system for modeling erythropoiesis of humans [7]. Zebrafish larvae were incubated with TRIAC for up to 24 h, and then, globin gene expression was assessed by quantitative real-time PCR (qPCR). The data showed that TRIAC administration strikingly increased *hbae5* mRNA levels, an ortholog of human *HBZ*, while had little effect on other embryonic globin genes expression (Fig. 1A). To further confirm this, we examined the effect of TRIAC on *hbae5* expression by whole-mount mRNA in situ hybridization (WISH). In line with the qPCR result, we found that the expression of *hbae5* was dramatically induced by TRIAC treatment (Fig. 1C).

**Fig. 1:**
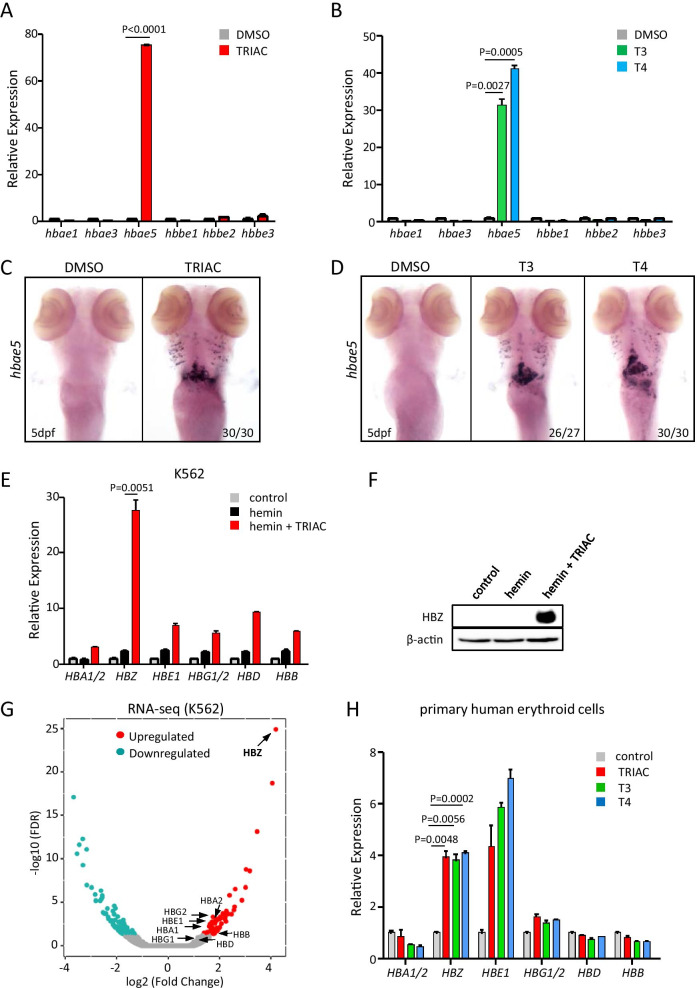
Thyroid hormones induce ζ-globin expression. **A**, **B** qPCR was performed on RNA from the TRIAC, T3- or T4-treated zebrafish embryos at 5 dpf and normalized to the level of zebrafish β-actin. Data shown are the means ± SEM. Statistical significance was calculated using the Student’s *t* test. **C**, **D** WISH assay of *hbae5* shows TRIAC, T3- or T4-induced ζ-globin gene expression in zebrafish embryos at 5 dpf, respectively. dpf, days post-fertilization. **E** TRIAC induced ζ-globin production in hemin-treated K562 cells. qPCR was performed after 48 h of incubation with TRIAC, and normalized to the level of human β-actin. Data shown are the means ± SEM. Statistical significance was calculated using the Student's *t* test. **F** Western blots of lysates of TRIAC-treated K562 cells. **G** Volcano plot of differentially expressed genes in TRIAC-treated K562 cells compared with control cells. Globin genes are indicated by arrows. FDR, false discovery rate. **H** Thyroid hormones induced ζ-globin gene expression in primary human erythroid cells. Human CD34^+^ cells were induced into an erythroid lineage and treated with TRIAC, T3 or T4, respectively. The total RNAs were isolated on day 7 of differentiation and subjected to qPCR analysis. Data shown are the means ± SEM. Statistical significance was calculated using the Student’s *t* test

Since the biological actions of TRIAC closely resemble those of the bioactive hormone 3,3′,5-triiodothyronine (T3) [8], we wonder whether T3 or the pro-hormone thyroxine (T4) has a similar effect. Zebrafish larvae were treated with T3 or T4 for up to 24 h, and then, qPCR and WISH assay were performed. Expectedly, the results showed that both T3 and T4 administration also significantly increase *hbae5* transcripts (Fig. 1B, D). Taken together, these data suggest that thyroid hormones have the abilities to selectively induce embryonic ζ-globin gene expression in zebrafish.

In order to examine whether thyroid hormone could also induce ζ-globin production in human cells, we used K562-derived erythroid cells. During hemin-induced erythroid differentiation of K562 cells, TRIAC was added and incubated for up to 48 h, and then, globin gene expression was assessed by qPCR assay and western blot. The results showed that TRIAC sharply increased *HBZ* mRNA and protein levels in hemin-treated K562 cells (Fig. 1E, F), although it also has a comparatively weak stimulatory effect on other globin genes expression. To evaluate genome-wide gene expression changes promoted by TRIAC, we performed RNA sequencing (RNA-seq) analysis. As expected, TRIAC-treated K562 cells showed significant induction of *HBZ*, with a mild induction in other globin levels (Fig. 1G). Thus, these experiments indicate that TRIAC preferentially induces ζ-globin expression in differentiated K562 cells.

We next determined whether thyroid hormone was able to induce ζ-globin expression in primary human erythroid cells. To this end, we used human CD34^+^ hematopoietic stem and progenitor cell (HSPC)-derived primary erythroblasts. During erythroid differentiation, CD34^+^ cells were incubated with TRIAC, T3 or T4, respectively. The total RNA samples were collected on day 7 of differentiation and then subjected to qPCR analysis. Again, we found that TRIAC, as well as T3 or T4, triggered an increase in *HBZ* mRNA levels, as compared with control cells (Fig. 1H). Collectively, these data suggest that thyroid hormones could also induce ζ-globin expression in human cells.

The biological effect of thyroid hormone is predominantly mediated by thyroid hormone receptors, which are encoded by the thyroid hormone receptor α (*THRA*) and thyroid hormone receptor β (*THRB*) genes. An increasing number of reports have implicated that THRA, but not THRB, is required for erythroid development [9–11], implying that ζ-globin expression might be positively regulated by THRA in erythroid cells. To determine whether THRA was essential for TRIAC-induced ζ-globin production, we first knocked down *THRA* expression in K562 cells by lentiviral-mediated short hairpin RNA (shRNA). As shown in Fig. 2A, the *THRA* shRNA efficiently reduced the *THRA* mRNA levels to 10% after 5 days of infection. Western blot using an anti-THRA antibody further confirmed that the THRA protein level was indeed decreased after knockdown of the *THRA* mRNA (Fig. 2B). Then, these shRNA-transduced K562 cells were treated with TRIAC for up to 48 h, and globin gene expression was assessed by qPCR. The result showed that THRA depletion reduced the level of *HBZ* mRNA by 40% compared to the control infected cells (Fig. 2C), indicating that THRA was involved in TRIAC-induced ζ-globin expression. We next examined TRIAC-induced ζ-globin expression level upon morpholino oligonucleotide (MO)-mediated thyroid hormone receptor α (*thraa*) knockdown in zebrafish. Zebrafish embryos were microinjected with specific translation-blocking morpholino against the *thraa*, homologous to human *THRA*, and exposed to TRIAC at 48 hpf. Then, embryos were collected for WISH assay after 24 h of TRIAC treatment. We found that Thraa deficiency reversed the increased expression of *hbae5* triggered by TRIAC (Fig. 2D). Taken together, these data indicated that THRA is the major effector responsible for TRIAC-induced ζ-globin expression.

**Fig. 2:**
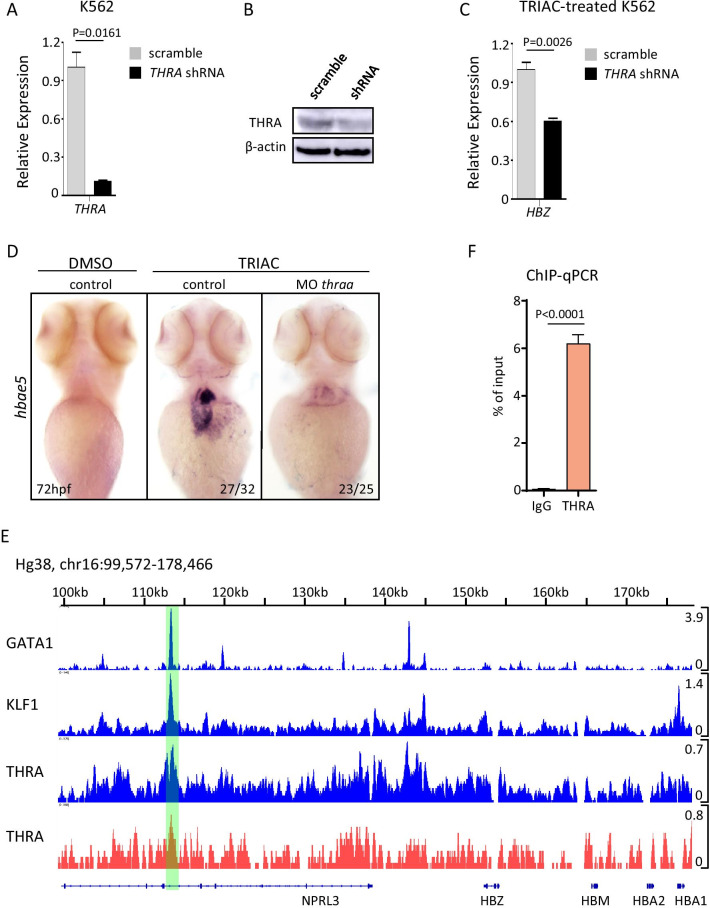
THRA inactivation reverses the elevated expression of ζ-globin triggered by TRIAC. **A** The shRNA-mediated knockdown of *THRA* results in a robust reduction of *THRA* mRNA level in K562 cells. Cells were infected with the indicated lentiviruses and then collected on the 5th day after viral infection for RNA analysis by qPCR. The lentiviral scrambled shRNA was used as the control. **B** *THRA* knockdown efficiency was assessed by Western blot using the anti-THRA antibody. β-actin: loading control. **C** *THRA* knockdown reduced the expression of ζ-globin in TRIAC-treated K562 cells. K562 stable lines expressing indicated shRNA were established and then collected after 48 h of incubation with TRIAC for RNA analysis by qPCR. **D** *Thraa* knockdown reduced the expression of ζ-globin in TRIAC-treated zebrafish embryos. Zebrafish embryos injected with the indicated MO were harvested after 24 h of TRIAC treatment for *hbae5* expression by WISH at 72 hpf. hpf, hours post-fertilization. **E** ChIP-seq signals at the α-like globin gene cluster (K562 cells) are shown. The peaks are highlighted in green. **F** ChIP-qPCR assay for THRA or IgG occupancy at HS-40 in K562 cells. Data shown are the means ± SEM. Statistical significance was calculated using the Student's *t* test

THRA is a member of the nuclear receptor superfamily and functions as a ligand-inducible transcription factor, which binds to thyroid hormone-responsive elements (TREs) to regulate gene transcription [12]. To determine whether THRA regulates ζ-globin expression directly or indirectly, we performed chromatin immunoprecipitation and sequencing (ChIP-seq) with an antibody to THRA in K562 cells. We observed that there was a moderate THRA ChIP-seq peak at ~ 40 kb upstream of the ζ-globin gene locus (Fig. 2E, red), which was known as HS-40, the most critical *cis*-regulatory element for α-like globin genes expression (Fig. 2E, blue) [13]. This finding is consistent with previously reported ChIP-seq data for THRA in K562 cells (Fig. 2E, blue) [14]. The ChIP-seq result was further validated by chromatin immunoprecipitation followed by quantitative PCR (ChIP-qPCR) (Fig. 2F). Thus, these data are highly likely to reflect authentic, direct binding sites of THRA in distal enhancer regulatory element.

In summary, this study shows that TRIAC emerges as a potent inducer of ζ-globin expression, which may allow development of new therapies for α-thalassemia or sickle-cell disease. Further studies need to investigate the in vivo potential of TRIAC in the treatment of these hemoglobin disorders.

## Supplementary Information

**Additional file 1.** Supplementary Materials and Methods.

**Additional file 2: Figure S1.** Morpholino efficacy assay. **A**, **B** Efficacy of *thraa* MO was tested by co-injection of the morpholinos together with GFP RNA containing the 5′UTR of the *thraa* gene into the embryo. GFP fluorescence was completely inhibited with full penetrance indicating that *thraa* morpholinos bind to their target sequence with high efficiency.

## Data Availability

The datasets used and/or analyzed during the current study are available from the corresponding author on reasonable request.

## Abbreviations

TRIAC: 3,3′,5-Triiodothyroacetic acid
THRA: Thyroid hormone receptor α
THRB: Thyroid hormone receptor β
qPCR: Quantitative real-time PCR
WISH: Whole-mount mRNA in situ hybridization
T3: 3,3′,5-Triiodothyronine
T4: Thyroxine
RNA-seq: RNA sequencing
HSPC: Hematopoietic stem and progenitor cell
shRNA: Short hairpin RNA
MO: Morpholino oligonucleotide
TREs: Thyroid hormone-responsive elements
ChIP-seq: Chromatin immunoprecipitation and sequencing

## References

[1] Modell B, Darlison M (2008). Global epidemiology of haemoglobin disorders and derived service indicators. Bull World Health Organ.

[2] Peschle C, Mavilio F, Care A (1985). Haemoglobin switching in human embryos: asynchrony of zeta––alpha and epsilon––gamma-globin switches in primitive and definite erythropoietic lineage. Nature.

[3] Russell JE, Liebhaber SA (1998). Reversal of lethal alpha- and beta-thalassemias in mice by expression of human embryonic globins. Blood.

[4] He Z, Russell JE (2004). Antisickling effects of an endogenous human alpha-like globin. Nat Med.

[5] Kunitake JM, Hartman N, Henson LC (1989). 3,5,3′-triiodothyroacetic acid therapy for thyroid hormone resistance. J Clin Endocrinol Metab.

[6] Groeneweg S, Peeters RP, Moran C (2019). Effectiveness and safety of the tri-iodothyronine analogue Triac in children and adults with MCT8 deficiency: an international, single-arm, open-label, phase 2 trial. Lancet Diabetes Endocrinol.

[7] Kulkeaw K, Sugiyama D (2012). Zebrafish erythropoiesis and the utility of fish as models of anemia. Stem Cell Res Ther.

[8] Groeneweg S, Peeters RP, Visser TJ, Visser WE (2017). Triiodothyroacetic acid in health and disease. J Endocrinol.

[9] Angelin-Duclos C, Domenget C, Kolbus A, Beug H, Jurdic P, Samarut J (2005). Thyroid hormone T3 acting through the thyroid hormone alpha receptor is necessary for implementation of erythropoiesis in the neonatal spleen environment in the mouse. Development.

[10] Kendrick TS, Payne CJ, Epis MR (2008). Erythroid defects in TRalpha−/− mice. Blood.

[11] Park S, Han CR, Park JW (2017). Defective erythropoiesis caused by mutations of the thyroid hormone receptor alpha gene. PLoS Genet.

[12] Cheng SY, Leonard JL, Davis PJ (2010). Molecular aspects of thyroid hormone actions. Endocr Rev.

[13] Jarman AP, Wood WG, Sharpe JA, Gourdon G, Ayyub H, Higgs DR (1991). Characterization of the major regulatory element upstream of the human alpha-globin gene cluster. Mol Cell Biol.

[14] Consortium EP (2012). An integrated encyclopedia of DNA elements in the human genome. Nature.

