# Supplementary material for: 3,3′,5-Triiodothyroacetic acid (TRIAC) induces embryonic ζ-globin expression via thyroid hormone receptor α
Source: J Hematol Oncol. 2021 Jun 26;14:99. doi: 10.1186/s13045-021-01108-z (PMC8235803; doi:10.1186/s13045-021-01108-z)
Supplement: Supplementary file 1 — Additional file 1. Supplementary Materials and Methods. [file 13045_2021_1108_MOESM1_ESM.docx]

Supplementary Materials for

**3,3',5-Triiodothyroacetic acid (TRIAC) induces embryonic ζ-globin expression via thyroid hormone receptor α**

Huiqiao Chen, Zixuan Wang, Shanhe Yu, Xiao Han, Yun Deng, Fuhui Wang, Yi Chen, Xiaohui Liu, Jun Zhou, Jun Zhu^*^ and Hao Yuan^*^

*Correspondence to: Dr. Jun Zhu (zhuj1966@yahoo.com) or Dr. Hao Yuan ([hyuan@sibs.ac.cn](mailto:hyuan@sibs.ac.cn)).

**This PDF file includes:**

Materials and Methods

Tables S1

References

**Materials and Methods**

**Zebrafish**

Zebrafish maintenance and staging were performed as described previously[1]. Zebrafish larvae were incubated with either 5 μM TRIAC (Selleckchem), 5 μM T3 (Sigma), 5 μM T4 (Sigma) or DMSO control for 24 hours, and fixed overnight at various stages of development in 4% paraformaldehyde at 4°C. Fixed embryos were washed in PBST (phosphate-buffered saline [PBS] with 0.1% Tween-20) and transferred into methanol for storage at -20°C. The zebrafish facility and study were approved by the Institutional Review Board of Shanghai Institutes of Biological Sciences, Chinese Academy of Sciences (Shanghai, China) and the methods were carried out in accordance with the approved guidelines.

**Whole-mount mRNA in situ hybridization (WISH)**

Digoxigenin-labeled antisense RNA probes were transcribed from linearized constructs using Sp6 polymerase. Whole-mount mRNA in situ hybridization was performed as described previously[2]. The probes were detected using alkaline phosphatase (AP)-coupled anti-digoxigenin Fab fragment antibody (Roche) with BCIP/NBT staining (Vector Laboratories).

**Quantitative real-time PCR (qPCR) and RNA sequencing (RNA-seq)**

Total RNA was extracted with TRIzol reagent (Invitrogen) according to the manufacturer's recommendations. cDNA was synthesized with the ReverTra Ace -α- kit (TOYOBO). qPCR was performed by a LightCycler 480 (Roche) following manufacturer's protocols. Relative expression was quantitated using the ΔΔCt method. The primers for qPCR were shown in Supplementary Table S1. RNA sequencing (RNA-seq) and data analysis were performed by Beijing igeneCode Biotech Co., Ltd.

**Western blot**

Western blot analysis was performed using standard methodology with the following antibodies: HBZ (R&D Systems, MAB7708), THRA (ORIGENE, TA805187) and β-actin (Cell Signaling Technology, 4970).

**Cell culture**

K562 cells were cultured in RPMI 1640 medium (Life Technologies) supplemented with 10% (v/v) fetal bovine serum at 37°C in humidified air with 5% CO2. Hemin (Sigma) was prepared as a 10 mM stock solution in 0.2 M NaOH and stored in aliquots at -20°C. 10 uM Hemin was used for erythroid differentiation of K562 cells. TRIAC, T3 or T4 was prepared as a 10 mM stock solution in DMSO and stored in aliquots at -20°C. 20 uM TRIAC, unless otherwise noted, was incubated with K562 cells for 48 hours.

Primary human CD34^+^ cells were obtained from umbilical cord blood (CB) mononuclear cells using positive immunomagnetic separation techniques by an EasySep™ Human CD34 Positive Selection Kit (STEMCELL Technologies) and were frozen down after isolation. CD34^+^ cells were cultured in a two-phase liquid culture system as described previously[3]. Briefly, CD34^+^ cells were maintained in expansion medium for a total of 6 days, and then were maintained in erythroid differentiation medium supplemented with either 5 uM TRIAC, 5 uM T3, 5 uM T4 or DMSO control. Cells were harvested on day 7 of differentiation. Umbilical cord blood was harvested at Rui Jin Hospital under an institutional review board-approved protocol.

**shRNA knockdown**

shRNA(5'CCGGGCGTAAGCTGATTGAGCAGAACTCGAGTTCTGCTCAATCAGCTTACGCTTTTTG3') targeting the THRA mRNA was acquired from MISSION shRNA (Sigma), and then cloned into pLKO.1-GFP vector. A scrambled shRNA was used as a control. Lentiviruses were prepared and infection of cells was carried out as described[4]. GFP^+^ cells were selected at 96 hours following infection by cell sorting using BD FACSAria III.

**Morpholino oligonucleotide (MO)**

MO against *thraa*[5] (5'CTCCTGCTCTGTGTTTTCCATTCAC3') was purchased from GeneTools. Zebrafish embryos were microinjected with MO at one-cell stage, and the dose of injection per embryo was 2.08 ng. Efficacy of *thraa* MO was tested by co-injection of the morpholinos together with GFP RNA containing the 5′UTR of the *thraa* gene into the embryo (Additional file 2: Figure S1).

**Chromatin immunoprecipitation (ChIP) assay**

ChIP assays were performed as previously described[6] with minor modiﬁcations. Briefly, K562 cells were harvested and crosslinked with 1% formaldehyde for 10 minutes at room temperature. After sonication, the soluble chromatins were incubated with the following antibodies separately: anti-THRA (ORIGENE, TA805187) or control IgG (Abcam, ab172730). Chromatin immunocomplexes were then precipitated with Protein G (Millipore, 16-662). The immunoprecipitated complex was washed, and DNA was extracted and purified by QIAquick PCR Purification Kit (QIAGEN, 28104). ChIP DNA was analyzed by qPCR, and the data were normalized by input DNA. The primers used for ChIP-qPCR are listed in Supplementary Table S1.

For ChIP-seq, extracted DNA was ligated to specific adaptors followed by deep sequencing in the Illumina HiSeq 4000 system as according to the manufacturer’s instructions. ChIP-Seq data was aligned to the human genome (hg38) reference genome using bwa version 0.7.10 with default parameter settings[7]. Subsequently, reads were filtered for duplicates and extended by 200bp. Visualization of read count data was performed by converting raw bam files to bigwig files using IGV tools[8] and normalized to 1 million reads. For the analysis of the ChIP-Seq datasets, we utilized MACS2 peak caller version 2.1.1 to identify peaks.

**ChIP-seq data analysis**

GATA1, KLF1 and THRA ChIP-seq data in K562 cells were reanalyzed from the previously published work[9-11]. ChIP-seq data were processed by Cistrome analysis pipeline and were loaded in UCSC genome browsers for visualization.

**Table S1. Primers of qPCR.**

| Gene |  | Sequence |
| --- | --- | --- |
| *β-actin* (*Danio rerio)* Forward Primer | real-time PCR | TGCTGTTTTCCCCTCCATTG |
| *β-actin* (*Danio rerio*) Reverse Primer |  | TTCTGTCCCATGCCAACCA |
|  |  |  |
| *hbae1* (*Danio rerio*) Forward Primer | real-time PCR | CTGAGGCTGTCAGCAAAATCG |
| *hbae1* (*Danio rerio*) Reverse Primer |  | GAACAAAGTGGCCAGAACCAC |
|  |  |  |
| *hbae3* (*Danio rerio*) Forward Primer | real-time PCR | GCTGATGGATGACCTGAAGGG |
| *hbae3* (*Danio rerio*) Reverse Primer |  | CTCAGGAGTGAAGTCGTCTGG |
|  |  |  |
| *hbae5* (*Danio rerio*) Forward Primer | real-time PCR | TGCTGAACCTCAGTGAATTGC |
| *hbae5* (*Danio rerio*) Reverse Primer |  | GGAACTTGTCGATGGCCAGAT |
|  |  |  |
| *hbbe1* (*Danio rerio*) Forward Primer | real-time PCR | TCCACGTAGATCCCGACAAC |
| *hbbe1* (*Danio rerio*) Reverse Primer |  | TACTGTCTTCCCAGAGCGGA |
|  |  |  |
| *hbbe2* (*Danio rerio*) Forward Primer | real-time PCR | GGACTGGACAGAGCCATGAAG |
| *hbbe2* (*Danio rerio*) Reverse Primer |  | GAGGCAATCACGATTGTCAGG |
|  |  |  |
| *hbbe3* (*Danio rerio*) Forward Primer | real-time PCR | TTGTGTGGACAGCTGAGGAG |
| *hbbe3* (*Danio rerio*) Reverse Primer |  | ACGGATAGACGACCAAGCAT |
|  |  |  |
| *β-actin* (*human*) Forward Primer | real-time PCR | CCAACCGCGAGAAGATGA |
| *β-actin* (*human*) Reverse Primer |  | CCAGAGGCGTACAGGGATAG |
|  |  |  |
| *HBA1/2* (*human*) Forward Primer | real-time PCR | AAGGTCGGCGCGCACGC |
| *HBA1/2* (*human*) Reverse Primer |  | CTCAGGTCGAAGTGCGGG |
|  |  |  |
| *HBZ* (*human*) Forward Primer | real-time PCR | GGACCATCATTGTGTCCATGT |
| *HBZ* (*human*) Reverse Primer |  | GGGAAGTAGGTCTTGGTCTGC |
|  |  |  |
| *HBE1* (*human*) Forward Primer | real-time PCR | TGCATGTGGATCCTGAGAAC |
| *HBE1* (*human*) Reverse Primer |  | CGACAGCAGACACCAGCTT |
|  |  |  |
| *HBG1/2* (*human*) Forward Primer | real-time PCR | AGCACCTGGATGATCTCAAG |
| *HBG1/2* (*human*) Reverse Primer |  | AAACGGTCACCAGCACATTT |
|  |  |  |
| *HBD* (*human*) Forward Primer | real-time PCR | GATGCAGTTGGTGGTGAGG |
| *HBD* (*human*) Reverse Primer |  | GGGTTGCCCATAACAGCAT |
|  |  |  |
| *HBB* (*human*) Forward Primer | real-time PCR | GCACGTGGATCCTGAGAACT |
| *HBB* (*human*) Reverse Primer |  | CACTGGTGGGGTGAATTCTT |
|  |  |  |
| *THRA* (*human*) Forward Primer | real-time PCR | TCCACATTGCCACAGAGG |
| *THRA* (*human*) Reverse Primer |  | GCATGGAGACAATGGGTGA |
|  |  |  |
| ChIP-qPCR Forward Primer | ChIP-qPCR | TACTGCTGATTACAACCTCT |
| ChIP-qPCR Reverse Primer |  | GCTCCAGATGAAGAACGTA |

**References**

1. Kimmel CB, Ballard WW, Kimmel SR, Ullmann B, Schilling TF. Stages of embryonic development of the zebrafish. Dev Dyn*.* 1995;203(3):253-310.

2. Bennett CM, Kanki JP, Rhodes J, et al. Myelopoiesis in the zebrafish, Danio rerio. Blood*.* 2001;98(3):643-651.

3. Sankaran VG, Menne TF, Xu J, et al. Human fetal hemoglobin expression is regulated by the developmental stage-specific repressor BCL11A. Science*.* 2008;322(5909):1839-1842.

4. Zhang YL, Sun JW, Xie YY, et al. Setd2 deficiency impairs hematopoietic stem cell self-renewal and causes malignant transformation. Cell Res*.* 2018;28(4):476-490.

5. Takayama S, Hostick U, Haendel M, Eisen J, Darimont B. An F-domain introduced by alternative splicing regulates activity of the zebrafish thyroid hormone receptor alpha. Gen Comp Endocrinol*.* 2008;155(1):176-189.

6. Yu SH, Zhu KY, Chen J, et al. JMJD3 facilitates C/EBPbeta-centered transcriptional program to exert oncorepressor activity in AML. Nat Commun*.* 2018;9(1):3369.

7. Li H, Durbin R. Fast and accurate short read alignment with Burrows-Wheeler transform. Bioinformatics*.* 2009;25(14):1754-1760.

8. Thorvaldsdottir H, Robinson JT, Mesirov JP. Integrative Genomics Viewer (IGV): high-performance genomics data visualization and exploration. Brief Bioinform*.* 2013;14(2):178-192.

9. Fujiwara T, O'Geen H, Keles S, et al. Discovering hematopoietic mechanisms through genome-wide analysis of GATA factor chromatin occupancy. Mol Cell*.* 2009;36(4):667-681.

10. Davis CA, Hitz BC, Sloan CA, et al. The Encyclopedia of DNA elements (ENCODE): data portal update. Nucleic Acids Res*.* 2018;46(D1):D794-D801.

11. Consortium EP. An integrated encyclopedia of DNA elements in the human genome. Nature*.* 2012;489(7414):57-74.
