# Supplementary figures and images for: 3,3′,5-Triiodothyroacetic acid (TRIAC) induces embryonic ζ-globin expression via thyroid hormone receptor α
Source: J Hematol Oncol. 2021 Jun 26;14:99. doi: 10.1186/s13045-021-01108-z (PMC8235803; doi:10.1186/s13045-021-01108-z)

## Supplemental Figure 1

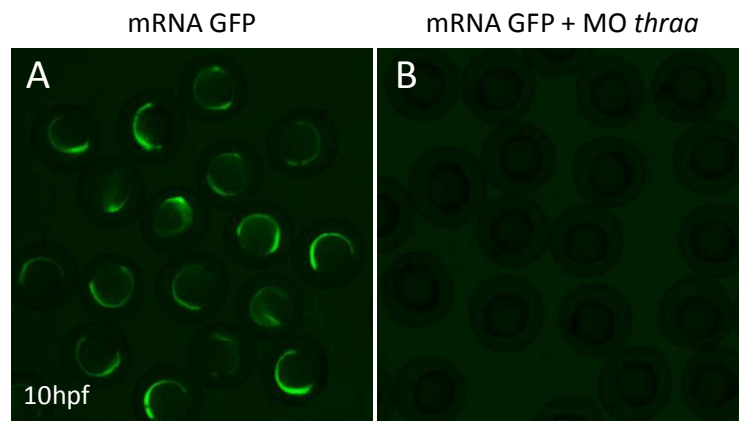

Supplement: Supplementary file 2 — Additional file 2: Figure S1. Morpholino efficacy assay. A, B Efficacy of thraa MO was tested by co-injection of the morpholinos together with GFP RNA containing the 5′UTR of the thraa gene into the embryo. GFP fluorescence was completely inhibited with full penetrance indicating that thraa morpholinos bind to their target sequence with high efficiency. [file 13045_2021_1108_MOESM2_ESM.pdf]
